# *PsicquicGraph*, a BioJS component to visualize molecular interactions from *PSICQUIC* servers

**DOI:** 10.12688/f1000research.3-44.v1

**Published:** 2014-02-13

**Authors:** Jose M. Villaveces, Rafael C. Jimenez, Bianca H. Habermann

**Affiliations:** 1 Max Planck Institute of Biochemistry, Am Klopferspitz 18, 82152, Germany; 2 European Bioinformatics Institute, Wellcome Trust Genome Campus, Hinxton, CB10 1SD, UK

## Abstract

**Summary:** Protein interaction networks have become an essential tool in large-scale data analysis, integration, and the visualization of high-throughput data in the context of complex cellular networks. Many individual databases are available that provide information on binary interactions of proteins and small molecules. Community efforts such as *PSICQUIC* aim to unify and standardize information emanating from these public databases. Here we introduce *PsicquicGraph*, an open-source, web-based visualization component for molecular interactions from *PSIQUIC* services.

**Availability:** *PsicquicGraph* is freely available at the BioJS Registry for download and enhancement. Instructions on how to use the tool are available here http://goo.gl/kDaIgZ and the source code can be found at http://github.com/biojs/biojs and DOI: 10.5281/zenodo.7709.

## Introduction

Proteins are one of the major actors in cellular processes and perform many different functions, which are required for the survival of a cell and an organism. Depending on the cell type, a different set of proteins will be available to ensure proper functioning of the cell within a larger context, for instance an organ. Typically, a cellular process is controlled by many different proteins that form a sophisticated network of interactions. Some proteins are even part of larger complexes, so-called molecular machines and the majority of interacting members are required to carry out a specific molecular task. In Systems Biology, we can use the networks of protein interactions to help us understand highly complex cellular processes.

Different efforts have been used to collect protein interactions. For example *IntAct*, an open-source, open data molecular interaction database ^1^ contains approximately 275 000 curated binary interactions extracted from over 5000 publications. *ChEMBL* is another example of an open source database ^2^ and holds more than 600 000 interactions between proteins and small molecules (chemicals).

In order to standardize access to interaction databases, the Proteomics Standard Initiative proposed the *Proteomics Standard Initiative Common QUery InterfaCe* ( *PSICQUIC*) ^3^ that defines:

   1. a web service with well defined methods to enable programmatic access to molecular interactions.

   2. a Molecular Interactions Query Language (MIQL ^4^), that specifies a syntax to allow flexible queries.

   3. a registry, that lists available *PSICQUIC* services and enables providers of databases for molecular interactions to register.

Meanwhile, 28 different databases have registered with *PSICQUIC*, including *IntAct* and *ChEMBL*, which altogether contain more than 150 million binary interactions.

Here, we present *PsicquicGraph*, a web component to visualize molecular interactions from *PSICQUIC* services. We have realized *PsicquicGraph* using BioJS ^5^, an open source JavaScript library of components for visualization of biological data on the web.

## The PsicquicGraph component

The minimal input for *PsicquicGraph* is (i) the URL of a valid *PSICQUIC* server, (ii) a valid MIQL query, (iii) a target container (HTML tag; usually a DIV) identifier to render the interactions graph and (iv) a proxy URL to bypass the same domain policy constraint in JavaScript.

**Figure 1.**
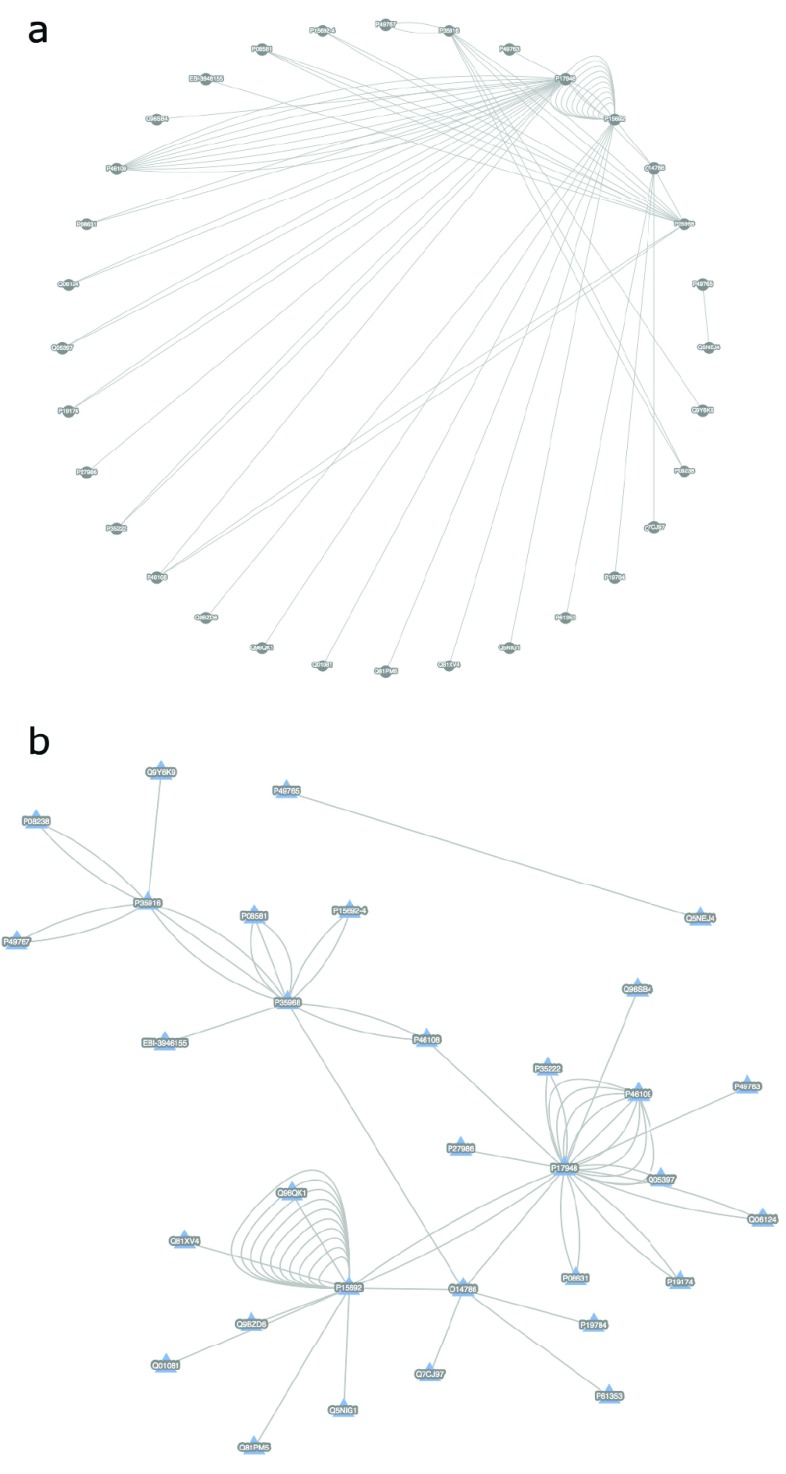
*PsicquicGraph* rendering of the proteins in the VEGF signaling pathway. The VEGF pathway is an essential regulator of vasculogenesis, as well as angiogenesis. In adults, it is up-regulated in a vast number of tumors. Solid tumors often build novel blood vessels and VEGF is one important regulator in that development. It is also a drug target in tumor medicine and several drugs directly target the VEGF receptor to block blood vessel formation in tumors. ( **a**) Default values were used to define the layout as well as other visualization options. ( **b**) A force-directed layout was used to render the graph and other visualization options such as *node shape*, *node color* and *node label* were customized.

Using the MIQL query, *PsicquicGraph* queries the *PSICQUIC* server. After retrieving the interactions in PSIMITAB ^6^ format, the interactions are parsed by *PsicquicGraph* and the graph is rendered using Cytoscape.js ^7^ ( Figure 1a).

The code below illustrates how to initialize *PsicquicGraph* by providing the minimal input. The *query* defined finds the first 100 human interactions (restricted by *maxResults*) and the *psicquicUrl* provided corresponds to the *IntAct* database. The name given to *target* constitutes the identifier of the component container.



                    var instance = new Biojs.PsicquicGraph ({
  target: 
                        
                            **ʼ**
                        example
                        
                            **ʼ**
                        , 
  psicquicUrl: 
                        
                            **ʼ**
                        http://www.ebi.ac.uk/Tools/
  webservices/psicquic/intact/webservices/
  current/search/query
                        
                            **ʼ**
                        , 
  proxyUrl: 
                        
                            **ʼ**
                        proxy.php
                        
                            **ʼ**
                        , 
  query: 
                        
                            **ʼ**
                        species:human? firstResult=0
  &maxResults=100
                        
                            **ʼ**
                        
});
                


By default, *PsicquicGraph* renders the graph using a circle layout. However, other layouts (force-directed, hierarchy, grid, random and preset) can be defined while initializing the component. Similarly, different visualization attributes such as *node shape*, *color* and *font family* can be defined ( Figure 1b).

## Conclusions

*PsicquicGraph* is a publicly available web component to render interactions from *PSICQUIC* servers. It relies on *PSICQUIC* and open data databases in order to simplify the rendering of complex protein-protein interaction networks.

The adoption of the BioJS specification facilitates *PsicquicGraph* integration, testing and documentation in addition to the potential exposure to new users.

## Software availability

Zenodo: PsicquicGraph, a BioJS component to visualize molecular inteactions from PSICQUIC servers, doi: 10.5281/zenodo.7709 ^8^.

GitHub: BioJS, http://github.com/biojs/biojs
