# Supplementary material for: PsicquicGraph, a BioJS component to visualize molecular interactions from PSICQUIC servers
Source: F1000Res. 2014 Feb 13;3:44. [Version 1] doi: 10.12688/f1000research.3-44.v1 (PMC4097353; doi:10.12688/f1000research.3-44.v1)
Supplement: Peer review history

# Peer review: Referee response for version 1

**DOI:** 10.5256/f1000research.3697.r4210

**Published:** 2014-03-25

**Authors:** Frederic de Lamotte, Jean-François Dufayard

**Affiliations:** 1 INRA, UMR AGAP, Montpellier, France; 2 CIRAD, Montpellier, France

This web tool article describes a component of BioJS, a javascript library of reusable components for the presentation of biological data. This particular tool, *PsicquicGraph*, is a web component intended to visualize molecular interactions from PSICQUIC servers.

PSICQUIC servers offer a standardised method for accessing proteomic interaction databases. From a biologists point of view, *PsicquicGraph *and the **BioJS initiative are extremely positive as it will allow for the retrieval of information from various databases in a seamless manner. 

Technically speaking, *PsicquicGraph* cleverly takes advantage of BioJS, Cytoscape.js as well as MILQ by providing useful interactions within all these modules. The links provided in this article allows access to decent documentation of the tool and also provides a useful working example.

**Improvements**

I consider that this article could be improved by listing the 28 databases that the authors claim to have registered with PSICQUIC and making them available in this article. That would allow the reader to easily figure out how important this standardization is.

I spent some times trying, but I was not successful in recreating the example in the window mode as advised by the authors (I tried Chrome, Firefox and Safari). I was thus not able to experience the demo example completely, which is bad for a web tool.

We have read this submission. We believe that we have an appropriate level of expertise to confirm that it is of an acceptable scientific standard.

# Peer review: Referee response for version 1

**DOI:** 10.5256/f1000research.3697.r3841

**Published:** 2014-02-24

**Authors:** Christine Orengo

**Affiliations:** 1 Institute for Structural and Molecular Biology, University College London, London, UK

This short article describes a component (PsicquicGraph) within the larger javascript library (BioJS) that assists the visualisation of molecular interactions on web pages. The component effectively provides the glue to stick the following pieces of technology together:

 - Cytoscape.js (a powerful tool for viewing networks on web pages)

 - MILQ (a language for querying molecular interactions)

 - BioJS (an event-driven javascript library which facilitates interaction between javascript widgets)

The component described in this article is already useful - mainly in that it provides a standard way of interacting with these existing modules and provides a single point of focus for future development. However there are some obvious developments the authors should make in a following version of the article that would make the component more useful:

1. Registering events (such as when a user selects a "node" or "edge").
2. Providing access to the underlying "cytoscape" javascript instance (to allow manipulation of the network after it has been created).

I have read this submission. I believe that I have an appropriate level of expertise to confirm that it is of an acceptable scientific standard.
